# Effect of Phenological Stage and Leaf Age on Changes of Chlorophyll and Carotenoid Contents in Some Weeds and Invasive Species

**DOI:** 10.3390/molecules30183788

**Published:** 2025-09-18

**Authors:** Kristina Ložienė, Ineta Chochlovaitė

**Affiliations:** 1 Pharmacy and Pharmacology Center, Institute of Biomedical Sciences, Faculty of Medicine, Vilnius University, M.K. Čiurlionio St. 21/27, 03101 Vilnius, Lithuania; inetachoch@gmail.com; 2 Laboratory of Economic Botany, State Scientific Research Institute Nature Research Centre, Akademijos St. 2, 08412 Vilnius, Lithuania

**Keywords:** chlorophyll *a*, chlorophyll *b*, chlorophyll *a*/*b* ratio, Apiaceae, Asteraceae, Lamiaceae, Rosaceae, Urticaceae

## Abstract

Potential sources of chlorophyll, widely used in the pharmaceutical and food industries, could be invasive species and weeds. The aim of the study was to estimate the effect of vegetation period and leaf age on changes of chlorophyll and carotenoid contents in leaves of six widespread plant species of four different families, as well as in the weed *Urtica dioica* as a “comparative” species. Plants were growing under the same environmental conditions, and chlorophylls and carotenoids were analyzed spectrophotometrically every two weeks from May to September. Average total chlorophyll (*a* + *b*) content only in leaves of *Lamium album* and *Aegopodium podagraria* was lower than in *Urtica dioica* and significantly differed from their content in *Leonurus cardiaca* and *Agrimonia eupatoria*. Total chlorophyll (*a* + *b*) content in leaves of common native *Pastinaca sativa* and very invasive *Solidago canadensis* was also higher or very similar to that of *Urtica dioica*. The highest amount of green pigments in all species was found at the budding and/or flowering period. Unlike other species, variation of total chlorophyll (*a* + *b*) in *Leonurus cardiaca* was little, and chlorophyll *a*/*b* ratio was lower than 2:1 during the vegetation period. In contrast to total chlorophyll (*a* + *b*), total carotenoid was higher in young leaves.

## 1. Introduction

Pigment chlorophyll, the most abundant compound on Earth, by collecting light energy during photosynthesis and converting it into chemical energy, plays not only the most important role in plants’ and algae’s biological processes but also has a wide spectrum of pharmacological properties [1,2,3]. In nature, there are more than 100 different chlorophyll structures, but of all the naturally occurring ones, chlorophyll *a* is the most widely distributed form, followed by chlorophyll *b*: these two green pigments are the predominant ones in higher plants at an approximately 3:1 ratio [1,2,4]. Although the leaf carotenoid profile (comprised mostly by photosynthetic unesterified carotenoids) is not abundant compared to the carotenoid amounts found in fruits and vegetables and is relatively constant, the transformation of these carotenoids during the leaf vegetation period is no less interesting from a plant physiological point of view than that of chlorophyll [5,6]. Plant leaf senescence, accompanied by changes in chlorophyll and carotenoid levels, is closely related to air temperature and active solar radiation [7,8,9]. However, the changes in these closely related pigments in leaves during their vegetative period may also depend on the plant species [10].

Chlorophyll and its different semi-synthetic metal-free and/or metallo-chlorophyll derivatives have not only various traditional therapeutic uses, including wound healing, anti-inflammatory agents, and internal deodorants, but research also demonstrated their antimicrobial, anti-mutagen, anti-carcinogen, and chemopreventive potential [8,11,12,13,14,15]. Probably the most widely used derivative from chlorophyll, water-soluble copper chlorophyllin is a widely used food supplement as a food coloring with “Generally Recognized As Safe” (GRAS) status; key applications for it are sugar confectionary, beverages, ice cream, water ices, fruit preparations, decorations and coatings, and sauces, and also specialized products such as Sage Derby cheese [15,16]. Both natural chlorophyll and its semi-synthetic derivatives, which are commercially produced by chemical modification of natural chlorophyll, are extracted from plants. Common sources of chlorophyll are leaf (spinach, parsley, and Brussels sprouts) and other (such as green beans, broccoli, green peas, and green paprika) vegetables, matcha green tea, alfalfa, and algae (chlorella and spirulina) [17]. Studies also demonstrate that fruit and vegetable wastes/by-products could be used for the extraction of chlorophyll and their further application as a colorant in food formulations [18]. However, to satisfy the large demand, new sources need to be sought. These could include plants that grow abundantly in natural habitats, sometimes even unwanted invasive plants or weeds. Such plant species are found in many plant families. For example, *Aegopodium podagraria* (Apiaceae), *Pastinaca sativa* (Apiaceae), and *Lamium album* (Lamiaceae) are often considered weeds due to their ability to spread aggressively and form abundant foliage [19,20,21]. *Solidago canadensis* (Asteraceae), a widespread invasive species throughout Europe, occupies large areas, overshadows native species, and is difficult to eradicate; therefore, its use could contribute to controlling its spread [22]. Also widely common, abundant in Europe, and large leaf biomass produces *Agrimonia eupatoria* (Rosaceae), as well as *Leonurus cardiaca* (Lamiaceae) and *Urtica dioica* (Urticaceae), which thrive in ruderal habitats [23,24,25]. The majority of these mentioned plants are also medicinal plants included in the European Pharmacopoeia (*L. cardiaca*, *A. eupatoria*, *S. canadensis*, and *U. dioica*) due to the biologically active compounds they accumulate.

Therefore, the above-mentioned widespread and common species of the Apiaceae, Asteraceae, Lamiaceae, and Rosaceae families were selected for the study, in leaves of which the dynamics of green pigments and carotenoids have not been studied or have been studied insufficiently, and which are often considered weeds or invasive species. As a “comparative” species, the widely common and considered ruderal species and weed *U. dioica* was chosen, the leaves of which are known to accumulate a high amount of chlorophyll.

## 2. Results

### 2.1. Changes in Total Chlorophyll (a + b) Content and Chlorophyll a/b Ratio During Plant Vegetation

The total chlorophyll (*a* + *b*) content of different plant species varied very unevenly during the vegetative period: from low variation in *L. cardiaca* (CV = 4%) to very high variation in *A. podagraria* and *L. album* (CV = 43% and CV = 39%, respectively) (Table 1).

During the vegetative period, the total chlorophyll (*a* + *b*) content in *U. dioica* leaves varied within the range of 1.00–2.21 mg/g. The highest total chlorophyll (*a* + *b*) content was determined from the intensive growth phase (mid-May) to the intensive flowering phase (second half of June) (Table 1). From the first half of July, the total chlorophyll (*a* + *b*) content decreased, and in the second half of this month, it was half as low as at the beginning of the vegetation period. In the first half of September, a total increase in the total chlorophyll (*a* + *b*) content was recorded in the studied *U. dioica* leaves to 1.92 mg/g, but after a few weeks, it decreased again. During the entire vegetative period, the amount of chlorophyll *a* was higher than that of chlorophyll *b*, and an increasing trend in the chlorophyll *a*/*b* ratio was also observed (Table 2).

Although the total chlorophyll (*a* + *b*) content of *A. podagraria*, compared to other studied species, varied within the lowest values (0.32–1.82 mg/g), its variation during the vegetative period was the highest (CV = 43%) (Table 1). The highest content of green pigments was determined in the phase of intensive growth and the buttonization phase. Later, the content of these pigments maintained a decreasing trend and reached the lowest content (almost 6 times lower than at the beginning of vegetation) at the beginning of September. In the middle of September, the total chlorophyll (*a* + *b*) content increased again (almost twice) compared to the beginning of September. Chlorophyll *a* dominated over chlorophyll *b* throughout the time in *A. podagraria* leaves, and the chlorophyll *a*/*b* ratio tended to increase and was twice as high at the end of the vegetative period as at the beginning of the vegetative period (Table 2).

The total chlorophyll (*a* + *b*) content in *P. sativa* leaves varied within the range of 1.43–2.22 mg/g. During the entire vegetative period, two decreases in the amount of green pigments were observed in the leaves of this species, in July and the second half of August. Although the amount of chlorophyll *a* in the leaves of *P. sativa* always dominated over the amount of chlorophyll *b*, unlike *A. podagraria*, the values of the chlorophyll *a*/*b* ratio varied more strongly throughout the vegetative season, and only in the second half of the vegetative period did the amount of chlorophyll *a* exceed the amount of chlorophyll *b* twice (Table 2).

In *L. album* plants, the highest total chlorophyll (*a* + *b*) content was determined at the beginning of the growth phase. Later, from the beginning of flower formation (second half of May), a gradual decrease in total chlorophyll (*a* + *b*) content was observed: in the buttonization phase, it was 16%; during, 30%; and after flowering, 31% lower than in the intensive growth phase. After the plants had finished flowering (from the second half of July), the total chlorophyll (*a* + *b*) content began to increase again (Table 1). Meanwhile, the total chlorophyll (*a* + *b*) content of *L. cardiaca*, another representative of the Lamiaceae family, varied (CV = 4%) the least among all the studied species during the entire vegetation period, and it was not established that its increase or decrease would have been statistically significant at any period (Table 1). Throughout the entire period of the study, chlorophyll *a* was almost twice as much as chlorophyll *b* in *L. album* leaves, and the increase in the chlorophyll *a*/*b* ratio coincided with the decrease in the total chlorophyll (*a* + *b*) content (Table 2). Also, a statistically significant positive correlation was established in *L. album* leaves between the amounts of chlorophyll *a* and chlorophyll *b* (r = 0.74, *p* < 0.05). Meanwhile, in the leaves of *L. cardiaca*, the amount of chlorophyll *a* either slightly exceeded the amount of chlorophyll *b* or was equal to it, and during intensive growth, even higher amounts of chlorophyll *b* than chlorophyll *a* were recorded (Table 2). Also, a weak but statistically significant (*p* < 0.05) negative correlation (r = −0.22) was found between chlorophyll *a* and chlorophyll *b* amounts in *L. cardiaca* leaves.

From the beginning of vegetation, the total chlorophyll (*a* + *b*) content in the leaves of *A. eupatoria*, a representative of the Rosaceae family, gradually increased, and the highest (2.72 ± 0.27 mg/g) was determined at the end of flowering (Table 1). A similar change in the total chlorophyll (*a* + *b*) content during the vegetative season was also observed in the leaves of *S. canadensis*, a representative of the Asteraceae family: the highest total chlorophyll (*a* + *b*) content in the leaves of this species was also determined during flowering (from the end of July to the beginning of September), and the lowest at the beginning of the vegetative season (Table 1). Chlorophyll *a* dominated in the leaves of *S. canadensis* throughout the entire period, exceeding the chlorophyll *b* content by as much as four times at the beginning of vegetation (Table 2). Also, a statistically significant (*p* < 0.05) moderate positive correlation (r = 0.49) was observed in the leaves of *S. canadensis* between the amounts of chlorophyll *a* and chlorophyll *b*. Unlike in the other studied species, in the leaves of *A. eupatoria* during buttonization and flowering, chlorophyll *a* was less than chlorophyll *b* in the total chlorophyll (*a* + *b*) fraction.

In all studied plants, the total chlorophyll (*a* + *b*) content was reliably negatively correlated with the chlorophyll *a*/*b* ratio, i.e., as the total chlorophyll (*a* + *b*) content in a plant increased, the amount of chlorophyll *a* decreased and chlorophyll *b* increased.

### 2.2. Changes in Total Carotenoid Content During Plant Vegetation

During the entire vegetation period, the total variation in the total carotenoid content in the plants of the studied species was large or very large, and in most cases it was larger compared to the variation in the total chlorophyll (*a* + *b*) content (Table 1 and Table 3). In all the studied species, the total carotenoid content at different times of the vegetation period differed significantly. *L. cardiaca* should be mentioned separately: while the total chlorophyll (*a* + *b*) content in its leaves varied little during the entire vegetation period (CV = 4%) and no significant changes were detected (Table 1), the total carotenoid content in the leaves of this species varied very strongly during the vegetation period (CV = 35%), and these changes in content were statistically significant (F = 4.83, *p* < 0.05) (Table 3).

Statistically significant (*p* < 0.05) negative (r = −0.27, r = −0.69, r = −0.65, and r = −0.48, respectively) correlations between total carotenoid and total chlorophyll (*a* + *b*) content were found in *P. sativa*, *L. cardiaca*, *S. canadensis*, and *A. eupatoria* leaves, while positive (r = 0.74 and r = 0.69, respectively) correlations were found in *A. podagraria* and *L. album* leaves.

### 2.3. Comparison of the Average Content of Total Chlorophyll (a + b) and Total Carotenoid Between Species, Leaves of Different Ages, Regardless of the Phenological Stage

**Figure 1:**
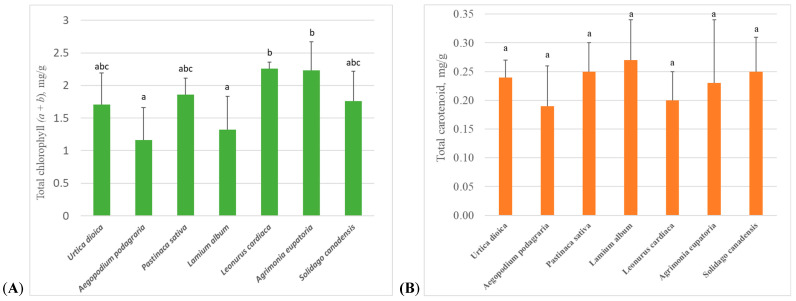
Average amount (mean ± standard deviation) of total chlorophyll (*a* + *b*) (**A**) and total carotenoid (**B**) in leaves of investigated plants, independent of phenological stage. Different and same letters above columns denote significant and not significant differences, respectively, between species (selected significant level *p* < 0.05).

The highest total chlorophyll (*a* + *b*) content, regardless of the phenological stage of the vegetation period, was found in the leaves of *A. eupatoria* and *L. cardiaca* (Figure 1A). The total chlorophyll (*a* + *b*) content in the leaves of plants of these species was almost twice as high and statistically significantly (*p* < 0.05) different from the content of these pigments in the leaves of *A. podagraria* and *L. album*, where it was the lowest among all the species analyzed in this study.

**Figure 2:**
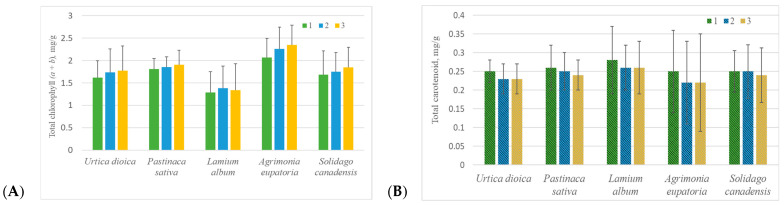
Average amount (mean ± standard deviation) of total chlorophyll (*a* + *b*) (**A**) and total carotenoid (**B**) in *Urtica dioica*, *Lamium album*, and *Solidago canadensis* simple leaves of different ages, as well as in *Pastinaca sativa* and *Agrimonia eupatoria* different leaflets of compound leaves, regardless of phenological stage (1—the first (the youngest) simple leaf from stem top or terminal leaflet of compound leaf; 2—the second simple leaf from stem top or the first lateral leaflet of compound leaf; 3—the third simple leaf from stem top or the second lateral leaflet of compound leaf). Kruskal–Wallis test demonstrated that total chlorophyll (*a* + *b*) as well as total carotenoid content did not differ significantly among the first, second, and third leaves or among different leaflets of compound leaves in any of the studied species.

In plants with simple leaves, the average total chlorophyll (*a* + *b*) content in the first leaves (the youngest) from the stem apex was the lowest compared to the second and the third leaves (Figure 2A). For example, in *U. dioica* and *S. canadensis*, the second and the third leaves contained 4–10% more chlorophyll, respectively, than the first leaf. A similar trend was observed in compound leaves of *P. sativa* and *A. eupatoria*: the second lateral leaflets also accumulated more chlorophyll than the terminal leaflets. However, no statistically significant differences in total chlorophyll (*a* + *b*) content were found among the first, second, and third leaves or among different leaflets of compound leaves.

**Figure 3:**
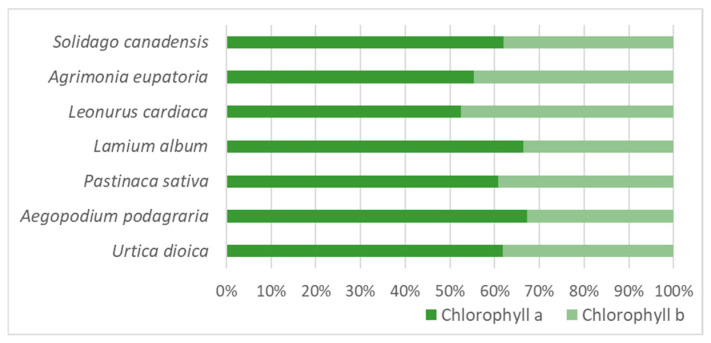
Percentage distribution of chlorophyll *a* and chlorophyll *b* in leaves, regardless of phenological stage, the position of the leaf on the stem, or the leaflet position in a compound leaf. The *t*-test showed that the chlorophyll percentage significantly (*p* < 0.05) differed from the chlorophyll *b* percentage in the leaves of all studied species.

In all studied species, regardless of the phenological stage and position of the leaf or leaflet on the stem or in the compound leaf, respectively, chlorophyll *a* was on average 1.1–2 times more than chlorophyll *b* (Figure 3). The lowest chlorophyll *a*/*b* ratio was found in the leaves of *L. cardiaca* and *A. eupatoria*: in them, the average amounts of chlorophyll *a* and chlorophyll *b* were very similar and differed by only 4% and 10%, respectively. Meanwhile, in the leaves of *A. podagraria* and *L. album*, chlorophyll *a* was twice as much as chlorophyll *b*.

The highest average total carotenoid content, regardless of phenological stage, was found in *L. album* leaves (0.27 ± 0.07 mg/g) (Figure 1B). The average content of these pigments was similar in *S. canadensis*, *P. sativa*, *U. dioica*, and *A. eupatoria* leaves, too, averaging 0.24 ± 0.01 mg/g, while the lowest was found in *L. cardiaca* and *A. podagraria* leaves, which were 26% and 30% lower than in *L. album* leaves, respectively. However, unlike total chlorophyll (*a* + *b*), the content of total carotenoid in leaves did not differ significantly between the studied plant species.

In contrast to total chlorophyll (*a* + *b*), in plants of all studied species, the amount of total carotenoid was highest in the youngest leaves and terminal leaflets (of simple and compound leaves, respectively), while in older leaves located further from the top of the stems and in lateral leaflets, the amount of these pigments gradually decreased (Figure 2B).

## 3. Discussion

It is known that *U. dioica* leaves are rich in chlorophyll [26]. However, the study found that, compared to other plants studied, the average total chlorophyll (*a* + *b*) content in *U. dioica* leaves was not the highest: in *U. dioica* leaves the total chlorophyll (*a* + *b*) content amounted to 1.71 ± 0.48 mg/g, and it was only 32% and 23% higher than in *A. podagraria* and *L. album* leaves, where the lowest total chlorophyll (*a* + *b*) contents were determined (Figure 1A). The highest total chlorophyll (*a* + *b*) content was observed in *A. eupatoria* and *L. cardiaca* leaves (Figure 1A). Although statistical analysis did not show a statistically significant difference in total chlorophyll (*a* + *b*) content between leaves of *U. dioica* and these two species, total chlorophyll (*a* + *b*) content in *A. eupatoria* and *L. cardiaca* leaves was 31% and 32% higher than in *U. dioica* leaves, respectively. Plants of all studied species were growing under the same environmental conditions and were maintained in the same way; therefore, differences in green pigment amounts between species could only depend on the genetic characteristics of the species. However, the amount of chlorophyll within a species can be influenced by environmental conditions, such as air temperature, solar radiation, etc. [2,9]. Therefore, this may be one of the reasons why, for example, the total chlorophyll (*a* + *b*) in leaves of *A. eupatoria*, growing wild in South Bulgaria and amounting to about 1.53 mg/g or 1.43 mg/g in dry or fresh (after recalculation, because the moisture of dry plants was indicated at 6.69%) leaves, respectively [27], amounted to only 64% of the total chlorophyll (*a* + *b*) content, averagely determined in our studied *A. eupatoria* leaves.

The dynamics of the total chlorophyll (*a* + *b*) content in the leaves during the vegetative season in the studied plants differed. The highest amounts of total chlorophyll (*a* + *b*) in the leaves of *A. podagraria*, *L. album*, and *U. dioica* were recorded during the intensive growth and flowering time, after which a tendency of decreasing total chlorophyll (*a* + *b*) content was observed during the entire remaining vegetative season. In Lithuania, these species (except *U. dioica*) bloom at the beginning of vegetation already from May [21,28]. Meanwhile, the total chlorophyll (*a* + *b*) content in the leaves of *P. sativa*, *A. eupatoria*, and *S. canadensis* gradually increased from the beginning of vegetation, reached the highest values during flower bud formation and/or the flowering period (June-July) (2.22 ± 0.12, 2.72 ± 0.27 mg/g, and 2.38 ± 0.13 mg/g, respectively), and then decreased again. Thus, although the dynamics of chlorophyll content during the vegetation season in the studied plants were different, the highest amounts of green pigments were recorded in all plants during the budding and/or flowering period. This may be influenced by the intensive synthesis processes of biologically active secondary metabolites occurring in plants at that time, since most of these metabolites in plants are usually detected during flowering. For example, the medicinal raw materials of *A. eupatoria* and *S. canadensis* (*Agrimoniae herba* and *Solidaginis herba*), included in the European Pharmacopoeia, are collected during the flowering period, since at that time the plants accumulate the most biologically active substances [29]. Of the seven plant species studied, *L. cardiaca* stood out with the most stable and least phenological stage-dependent total chlorophyll (*a* + *b*) content: although the highest total chlorophyll (*a* + *b*) content was recorded during intensive growth (May), the variation in its content throughout the vegetative season was very small (CV = 4%) and, unlike in other species, statistically insignificant (Table 1).

Regardless of the phenological stage, in the leaves of the studied plant species, chlorophyll *a* constituted a larger part of the total chlorophyll (*a* + *b*), but only in the leaves of *A. podagraria* and *L. album* did the chlorophyll *a*/*b* ratio reach 2:1 (Figure 3). In the leaves of *L. cardiaca*, the average chlorophyll *a*/*b* ratio was the lowest (Figure 3), and, unlike in the other studied species, it never reached 2:1 during the entire vegetative period, and a higher amount of chlorophyll *b* than chlorophyll *a* was determined at the beginning of the vegetative period (at the stage of vegetative growth) (Table 2). The literature indicates that chlorophyll *a* and chlorophyll *b* dominate in higher plants, and their ratio is usually 3:1 [2,12]. In the leaves of most of the studied species, a lower chlorophyll *a*/*b* ratio was observed at the beginning of the vegetative period, and then it gradually increased and was highest in the second half of the vegetative period; and only in the leaves of *A. eupatoria* and *S. canadensis* was the highest chlorophyll *a*/*b* ratio recorded at the beginning of the vegetation, and then it maintained a decreasing trend (Table 2). It is known that the ratio of these chlorophylls depends on the plant species and agroclimatic conditions [2,12,30]. The increasing amount of chlorophyll *b* in plants broadens the spectrum of absorbed light waves; therefore, in low light conditions, plants produce a higher amount of chlorophyll *b* [31]. Also, both chlorophylls differ in their thermal stability: chlorophyll *b* is thermally more stable than chlorophyll *a* [2]. However, during the study, the plants of all studied species were cared for in the same way and were exposed to the same environmental conditions (for example, they all grew in a sunny, unshaded place in the same temperature regime); therefore, the different changes in the chlorophyll *a*/*b* ratio during the vegetative period in different species could be influenced by the genetic characteristics of the species, which in turn demonstrates that the dynamics of the chlorophyll *a*/*b* ratio in different plant species differ. Chlorophyll *a* has a stronger antioxidant activity than chlorophyll *b* [8]. Therefore, when collecting plant raw materials for chlorophyll extraction and in order to obtain chlorophyll with higher antioxidant activity, it is useful not only to know in which phenological stage the chlorophyll *a*/*b* ratio is the highest but also to take into account the fact that a reliable negative correlation has been established between the chlorophyll *a*/*b* ratio and the total chlorophyll (*a* + *b*) content.

Carotenoids, which include many biologically active and pharmacologically valuable compounds, do not accumulate in large quantities in plant leaves, but they also participate in harvesting light energy for photosynthesis [6]. Therefore, carotenoids were of interest in this study because of their relationship with chlorophylls from the physiological side of plants. On average, carotenoids were 5–11 times less than total chlorophyll (*a* + *b*) in the studied plants (Figure 1). During the vegetation period, as the total chlorophyll (*a* + *b*) content decreased, the carotenoid content in plants increased (Table 1 and Table 3), and the negative correlation between the content of these pigments was statistically significant (*p* < 0.05). This is related to the seasonal variability of physiological processes in plants: as the plant’s growing season approaches its end, leaves undergo senescence, and photosynthesis slows down, as a result of which chlorophyll gradually decreases and the carotenoid content increases [9]. However, this phenomenon was not observed in all studied species: in the leaves of *A. podagraria* and *L. album*, a statistically significant (*p* < 0.05) positive correlation was found between the total carotenoid and total chlorophyll (*a* + *b*) content. It is also interesting that the total carotenoid content was higher and the total chlorophyll (*a* + *b*) content was lower in leaves closer to the top of the stem, where they are the youngest and most vulnerable, compared to older leaves (Figure 2A,B). It is known that carotenoids protect plant cells and tissues from photooxidative damage, participate in a light protection mechanism during excessive light incidence, and are precursors of plant hormone abscisic acid, which is involved in the maturation and differentiation of cells and in tolerance to abiotic stress [32]. All of this is especially relevant for young, developing, and growing leaves.

## 4. Materials and Methods

### 4.1. Plant Material

The plants of *L. cardiaca*, *A. eupatoria*, *S. canadensis*, and *P. sativa* cultivated in open ground in the field collection of the State Research Institute Nature Research Center (Mažieji Gulbinai, Vilnius, Lithuania; 54°46′ N, 25°17″ E), as well as the plants of *L. album*, *U. dioica*, and *A. podagraria* growing naturally there, were studied. During the study, all plants grew under the same environmental conditions, were equally illuminated by the sun, and were not fertilized or watered. The leaves for the studies from these plants were collected every two weeks from May to September (the exact collection dates are given in Table 1, Table 2 and Table 3). The first (the youngest), the second, and the third leaf from the tops of the main stem were collected and analyzed separately in *U. dioica*, *L. album*, and *S. canadensis* plants; also, the terminal, the first, and the second lateral leaflets of the top compound leaf of the main stems were collected and analyzed separately in *A. eupatoria* and *P. sativa* plants.

### 4.2. Sample Preparation and Determination of Pigment Content

To determine the pigment content, three samples of 0.04–0.05 g were taken from each fresh leaf. Each leaf sample was filled separately with 2 mL of N, N-dimethylformamide (≥99.8%, Honeywell, St. Louis, LA, USA) and stored in a refrigerator for two weeks at 4 °C. The amounts of chlorophyll *a*, chlorophyll *b*, and total carotenoid, as well as total chlorophyll (sum of chlorophyll *a* and chlorophyll *b*), were determined using a Biochrom Libra S32PC spectrophotometer (Cambridge, UK); the absorption of each extract was measured at 470 nm, 648 nm, and 664 nm, and N, N-dimethylformamide was used as a control. The concentrations of different pigments were calculated using the formulas presented by A. R. Wellburn (1994) [33].

### 4.3. Statistical Analysis

Statistical data processing, including calculation of means, standard deviations, one-way ANOVA, the Kruskal–Wallis test, the *t*-test, the H values, and Pearson rank correlations, was carried out with STATISTICA^®^ 10 and Microsoft Excel 2010. The probability level was *α* = 0.05.

## 5. Conclusions

As it was seen, the highest amounts of total chlorophyll (*a* + *b*), regardless of the vegetation period and leaf age, accumulate in *L. cardiaca* and *A. eupatoria* leaves. These two medicinal plants are native, frequent, and common not only in the Baltic region but also throughout Europe and could be potential sources of natural chlorophyll. Since the total chlorophyll (*a* + *b*) content variation in *L. cardiaca* leaves during the entire vegetation period was low, it can be assumed that the raw material of *L. cardiaca* grass for chlorophyll extraction could be collected regardless of the phenological phase of the plant. Meanwhile, it would be appropriate to collect biomass of *A. eupatoria* leaves for chlorophyll extraction during flowering. Also worth noting is *S. canadensis*, the average total chlorophyll (*a* + *b*) content in the leaves of which was the same as in the leaves of *U. dioica.* *S. canadensis* is not only a medicinal plant included in the European Pharmacopoeia but also an invasive plant in Europe. Therefore, the raw material of *S. canadensis* leaves can be collected without restrictions. It would only be appropriate to note that although total chlorophyll (*a* + *b*) is most abundant in *S. canadensis* leaves during flowering, the chlorophyll *a*/*b* ratio is lowest at that time, i.e., at that time the amounts of chlorophyll *a* and chlorophyll *b* in the chlorophyll fraction are equal.

## Figures and Tables

**Table 1:** Changes of total chlorophyll (*a* + *b*) amount (mean ± standard deviation) during the vegetation period (CV—coefficient of variation; *—effects are significant at *p* < 0.05).

Species	Total Chlorophyll (*a* + *b*) (mg/g) at Different Times of the Vegetation Period (Month/Day)										CV (%)	H-Value
	12 May	26 May	9 June	23 June	7 July	21 July	4 August	18 August	1 September	15 September		
*Aegopodium podagraria*	1.82 ± 0.05	1.64 ± 0.06	1.74 ± 0.23	1.15 ± 0.08	1.46 ± 0.11	1.04 ± 0.08	1.05 ± 0.20	0.79 ± 0.16	0.32 ± 0.01	0.60 ± 0.27	43	66.1 *
*Pastinaca sativa*	1.86 ± 0.09	1.98 ± 0.04	2.14 ± 0.04	2.22 ± 0.12	1.76 ± 0.09	1.54 ± 0.08	2.01 ± 0.15	1.43 ± 0.13	1.70 ± 0.03	1.92 ± 0.21	13	40.7 *
*Lamium album*	2.02 ± 0.16	1.69 ± 0.06	1.42 ± 0.06	1.40 ± 0.07	0.54 ± 0.08	0.79 ± 0.06	1.35 ± 0.05	–	–	–	39	49.7 *
*Leonurus cardiaca*	2.33 ± 0.13	2.42 ± 0.05	2.21 ± 0.02	2.29 ± 0.03	2.24 ± 0.11	2.28 ± 0.07	2.15 ± 0.04	2.24 ± 0.11	2.32 ± 0.15	2.07 ± 0.07	4	14.8
*Agrimonia eupatoria*	1.66 ± 0.17	1.67 ± 0.13	2.42 ± 0.16	2.40 ± 0.18	2.61 ± 0.09	2.50 ± 0.06	2.72 ± 0.27	2.44 ± 0.38	2.31 ± 0.30	1.52 ± 0.05	18	46.0 *
*Solidago canadensis*	0.98 ± 0.17	1.37 ± 0.07	1.50 ± 0.29	1.75 ± 0.12	1.41 ± 0.06	2.38 ± 0.04	2.38 ± 0.13	2.08 ± 0.08	2.07 ± 0.04	1.66 ± 0.05	26	68.9 *
*Urtica dioica*	2.21 ± 0.09	2.21 ± 0.21	2.18 ± 0.28	2.14 ± 0.13	1.74 ± 0.16	1.00 ± 0.05	1.27 ± 0.18	1.28 ± 0.16	1.92 ± 0.04	1.28 ± 0.05	27	67.0 *

**Table 2:** Changes of chlorophyll *a*/*b* ratio (mean ± standard deviation) during the vegetation period (CV—coefficient of variation; *—effects are significant at *p* < 0.05).

Species	Chlorophyll *a*/*b* Ratio at Different Times of the Vegetation Period (Month/Day)										CV (%)	H-Value
	12 May	26 May	9 June	23 June	7 July	21 July	4 August	18 August	1 September	15 September		
*Aegopodium podagraria*	1.7 ± 0.5	2.3 ± 0.2	1.8 ± 0.4	2.3 ± 0.2	1.9 ± 0.3	2.7 ± 0.3	2.5 ± 0.2	2.4 ± 0.1	2.7 ± 0.4	2.7 ± 0.4	22	52.0 *
*Pastinaca sativa*	1.7 ± 0.3	1.7 ± 0.2	1.3 ± 0.2	1.2 ± 0.2	1.9 ± 0.5	2.1 ± 0.3	1.5 ± 0.7	2.1 ± 0.4	2.2 ± 0.8	1.9 ± 0.5	33	38.4 *
*Lamium album*	1.5 ± 0.4	2.1 ± 0.3	2.4 ± 0.2	2.3 ± 0.5	2.3 ± 0.4	2.6 ± 0.2	1.9 ± 0.3	–	–	–	27	30.9 *
*Leonurus cardiaca*	1.2 ± 0.4	0.9 ± 0.2	1.3 ± 0.4	1.0 ± 0.2	1.2 ± 0.3	1.1 ± 0.1	1.2 ± 0.5	1.0 ± 0.2	1.3 ± 0.5	1.5 ± 0.3	33	27.4 *
*Agrimonia eupatoria*	2.5 ± 0.4	2.3 ± 0.6	1.0 ± 0.2	0.9 ± 0.3	0.8 ± 0.2	0.8 ± 0.3	1.3 ± 0.5	1.3 ± 0.5	2.0 ± 0.4	2.1 ± 0.3	47	56.1 *
*Solidago canadensis*	4.1 ± 0.7	3.0 ± 0.5	2.4 ± 0.8	2.0 ± 0.2	2.6 ± 0.4	1.0 ± 0.1	1.2 ± 0.5	1.3 ± 0.6	1.4 ± 0.3	2.5 ± 0.7	46	66.7 *
*Urtica dioica*	1.3 ± 0.5	1.2 ± 0.3	1.3 ± 0.4	1.3 ± 0.3	2.0± 0.7	2.8 ± 0.7	3.0 ± 0.4	2.8 ± 0.5	1.7± 0.2	3.0 ± 0.5	43	66.1 *

**Table 3:** Changes of total carotenoid amount (mean ± standard deviation) during the vegetation period (CV—coefficient of variation; *—effects are significant at *p* < 0.05).

Species	Total Carotenoid (mg/g) at Different Times of the Vegetation Period (Month/Day)										CV (%)	H-Value
	12 May	26 May	9 June	23 June	7 July	21 July	4 August	18 August	1 September	15 September		
*Aegopodium podagraria*	0.27 ± 0.00	0.31 ± 0.01	0.17 ± 0.02	0.20 ± 0.01	0.21 ± 0.01	0.19 ± 0.01	0.20 ± 0.03	0.15 ± 0.03	0.08 ± 0.00	0.11 ± 0.03	35	64.4 *
*Pastinaca sativa*	0.29 ± 0.03	0.30 ± 0.00	0.23 ± 0.02	0.21 ± 0.00	0.23 ± 0.01	0.26 ± 0.01	0.15 ± 0.03	0.27 ± 0.01	0.26 ± 0.02	0.32 ± 0.03	24	54.3 *
*Lamium album*	0.31 ± 0.07	0.37 ± 0.03	0.29 ± 0.03	0.31 ± 0.03	0.18 ± 0.05	0.21 ± 0.03	0.21 ± 0.04	–	–	–	30	46.2 *
*Leonurus cardiaca*	0.22 ± 0.03	0.15 ± 0.03	0.25 ± 0.03	0.15 ± 0.02	0.26 ± 0.01	0.17 ± 0.02	0.18 ± 0.00	0.17 ± 0.02	0.20 ± 0.02	0.27 ± 0.02	35	29.8 *
*Agrimonia eupatoria*	0.33 ± 0.00	0.33 ± 0.01	0.17 ± 0.05	0.13 ± 0.08	0.13 ± 0.03	0.15 ± 0.03	0.12 ± 0.03	0.18 ± 0.03	0.40 ± 0.04	0.37 ± 0.02	48	60.2 *
*Solidago canadensis*	0.29 ± 0.04	0.32 ± 0.02	0.26 ± 0.03	0.30 ± 0.01	0.28 ± 0.00	0.15 ± 0.02	0.17 ± 0.07	0.19 ± 0.02	0.24 ± 0.02	0.31 ± 0.01	32	52.6 *
*Urtica dioica*	0.22 ± 0.01	0.22 ± 0.05	0.21 ± 0.03	0.21 ± 0.03	0.24 ± 0.02	0.19 ± 0.00	0.28 ± 0.02	0.26 ± 0.01	0.27 ± 0.00	0.29 ± 0.02	21	43.2 *

## Data Availability

Data are contained within the article.
